# Inside the Mouths of Caregivers: Evaluating Oral Health Status of Iranian Female Dental Nurses—A Pilot Study Findings

**DOI:** 10.1155/ijod/7818864

**Published:** 2025-09-18

**Authors:** Ehsan Babaei Zarch, Seyed Mohammad Heidarpour Zarchi, Fatemeh Owlia

**Affiliations:** ^1^Department of Oral and Maxillofacial Medicine, School of Dentistry, Shahid Sadoughi University of Medical Sciences, Yazd, Iran; ^2^School of Dentistry, Shahid Sadoughi University of Medical Sciences, Yazd, Iran

**Keywords:** dental, education, nurses, oral health, oral health behavior, oral hygiene habits

## Abstract

**Background:** Poor oral health is also a notable cause of pain and discomfort and is usually a pointer to general health. Dental nurses play a significant role in improving the oral health of the community. This study presents the assessment and evaluation of the oral health status of Iranian dental nurses based on the oral health questionnaire (OHQ) developed by the WHO.

**Materials and Methods:** This descriptive cross-sectional survey studied 50 informally trained dental nurses. Subjects were recruited through a census sampling technique from two dental clinic centers affiliated with Shahid Sadoughi University of Medical Sciences, Yazd, Iran, between August and October 2023. Oral health status and oral health behavior were measured using the WHO OHQ for dental nurses.

**Results:** The mean age of participants was 35.52 ± 7.86 years. All respondents reported brushing their teeth and 48 (96%) used toothpaste. Similarly, 95% of participants reported having a total of 20 or more teeth. Of the sample, 44% had visited the dentist in past 6 months. Singles had significantly better oral health behavior scores than married subjects and also showed fewer signs of oral and dental health problems. The mean scores of oral problems did not differ significantly by age, systemic diseases, work experience, workplace, or household family income.

**Conclusion:** Generally, the majority of Iranian dental nurses in the study assessed their oral health status as good, while their scores concerning oral health behaviors were very poor. Emphasizing the importance of academic educational courses for dental nurses is crucial in promoting oral health behavior. This study demonstrated that merely having easy access to dental services is not a sufficient replacement for formally institutionalized training in dental nurses.

**Summary**

• **Research gap and novelty statement**
  • Lacking previous studies: To our knowledge, this study is the first to provide a comprehensive assessment of oral health behaviors and challenges among Iranian dental nurses. Likely, the study group was nonformally trained about oral and dental problems. This would imply a wide gap in the available information on oral health practices and consequences in the group that forms the nucleus of health provision in Iran.
• **Novelty of the present study:**
  • Unique focus on dental nurses: The current study will contribute to the literature gap regarding oral health behaviors and problems in dental nurses in Iran, a developing country, who have often been neglected in most previous studies. Focusing particularly on a research population will provide insight into their specific oral health needs and issues. This study's findings can inform the development of interventions to improve oral health among dental nurses. The significance of studying this group lies in its easy accessibility and relatively low cost compared with other populations.

## 1. Introduction

Oral hygiene is considered a major aspect of general health. The study has pointed out an association between oral health and oral diseases with systemic health consequences, such as diabetes, gastrointestinal diseases, cardiovascular diseases, and autoimmune diseases [1]. This simply shows that the mouth's condition reflects the body's overall health. Poor health can, therefore, become a threat to general well-being due to neglect of the overall health condition [2]. Oral diseases are a source of severe global health and economic burdens [3]. Self-assessment of oral health status is the symbol of a person's perception of the need to visit a dentist. Despite the possibility of preventing oral diseases, their prevalence remains high, with approximately 3.5 billion cases worldwide [4]. An unhealthy diet, sedentary lifestyle, tobacco and alcohol abuse, mental stress, and poor hygiene practices contribute to poor oral and dental hygiene. Besides the socioeconomic aspect, a poor understanding of health further restricts dental care service utilization and proper oral care practices. These are barriers that reduce oral health maintenance. Nurses as healthcare providers are mandated to share knowledge in promoting health. They support health-related activities, encourage healthy lifestyles and behaviors, and develop knowledge, attitudes, and behaviors that are conducive to maintaining good oral health and preventing oral diseases [5]. Self-assessment of oral health behaviors mainly encompasses the assessment of the frequency of brushing, techniques of brushing, use of dental floss or mouthwash, visits to dentists, and oral and dental hygiene product use [3, 6, 7]. A study about self-assessment on oral and dental health behaviors of medical students indicated that first-year dental students showed significant improvement as compared with the medical students regarding frequency and proper techniques of brushing [7]. A study showed significant variation between the two genders concerning oral health behaviors, as it was worse in the male population, dental visits are less and oral hygiene habits are poor [8, 9]. Past studies have shown that most nurses possess insufficient knowledge about oral health [10–12]. Several studies have demonstrated that nonformally trained nurses have good basic knowledge of oral health, but its knowledge about the relationship between systemic diseases and the importance of the oral cavity was insufficient [1, 13]. A few reports have been published about oral health status and behaviors in dental nurses and their correlation with demographic variables.

## 2. Methods

### 2.1. Study Design and Population

This cross-sectional study focused on 50 female dental nurses recruited through a convenience sampling method. The eligibility criteria for participants included:

a. working in governmental dental clinics affiliated with Shahid Sadoughi University of Medical Sciences, Yazd, between August 2023 and October 2023;
b. being aged between 18 and 65 years;
c. having no formal training related to dental nursing;
d. being willing to participate in the study. Written informed consent was obtained from each participant.

### 2.2. Data Collection

The data for this study were collected using the standard WHO's oral health questionnaire (OHQ) for adults to enhance standardization, which was translated and psychometrically evaluated in Akbari et al.'s [14] research. The instrument's validity and reliability were confirmed, with a Cronbach's *α* coefficient of 0.86 for oral health status items and behaviors and 0.91 for problem items [14]. It was used to measure the oral health status and oral health behaviors of dental nurses. To collect data, a printed questionnaire was prepared and distributed to the participants. At the start of the research, the participants were given an explanation of the research process and goals. They were then given the option to complete the questionnaire after receiving all relevant information. All provided information was handled with the highest level of confidentiality and anonymity.

### 2.3. Data Analysis

This questionnaire includes 29 items grouped into three axes. Initially, demographic information was gathered. The questionnaire contained a total of 19 items in two sections. Ten items focus on oral and dental health status, while the remaining nine evaluate oral and dental health behaviors. To assess oral health status, four questions were utilized: (a) self-reported number of teeth present, with choices from 0 (no natural teeth) to 3 (20 natural teeth or more); (b) experience of oral/dental pain or discomfort (yes/no); (c) use of removable dentures, with options for partial, full upper, or full lower (yes/no); (d) self-assessment of the condition of teeth and gums, rated on a scale from 1 (excellent) to 6 (very poor).

Three items were asked to assess oral health behaviors: (a) the frequency of performing oral hygiene, ranging from never (1) to once a day (6); (b) the use of aids/tools for oral hygiene, such as a toothbrush, wooden toothpick, and dental floss, with options of yes or no; (c) the use of fluoride or non-fluoride toothpaste, with options of yes or no. The oral and dental health behavior score was determined based on the scores of these nine items, with a minimum score of zero and a maximum score of 9. A score of 5 or lower indicated poor behavior, a score of 6–7 indicated moderate behavior, and a score of 8–9 suggested good behavior. There are two questions regarding dental visits. The scores range from 1 (less than 6 months ago) to 5 (5 years ago or longer). Another question was asked about the reasons for the visits, such as consultation/advice, treatment/follow-up, or dental/oral pain/discomfort.

In the third part, oral health problems were assessed via 10 yes/no questions. Each “yes” response was assigned one point, while each “no” response received zero points. The problem scores ranged from zero to 10. Concerning the “problem” score, a range of 0–5 indicated few problems, a range of 6–7 indicated moderate issues, and a score of 8 or higher indicated numerous problems.

### 2.4. Ethical Considerations

This study was conducted after approval was obtained from the Committee of Ethics in Human Research at Shahid Sadoughi University of Medical Sciences, Yazd. The code of ethics for this study is IR.SSU.DENTISTRY.REC.1402.008.

### 2.5. Statistical Analysis

After the data were collected, data analysis was done via SPSS version 27. To assess the normality of the quantitative variables, the Shapiro–Wilk test was used. Since the quantitative variables were nonnormally distributed (*p*-value < 0.05), nonparametric tests, such as the *T*-test, the ANOVA test, and the Spearman correlation coefficient, were used for data analysis. A significance level of 0.05 was considered.

## 3. Results

A total of 50 female dental nurses with a mean age of 35.52 ± 7.86 years and a range of 20–54 years were included in this study.

Of the participants, 45 (90%) of the nurses had 20 or more teeth in their mouths. Nineteen nurses (38%) considered their teeth's health status good, and 24 nurses (48%) stated that their gums were good. Among all dental nurses surveyed, 80% reported regular use of fluoride toothpaste, while all participants indicated daily toothbrushing, and only 38% reported daily flossing. Regarding removable prostheses, two nurses (4%) had partial maxillary dentures, one nurse (2%) had partial mandibular dentures, and one of the dental nurses (2%) had partial dentures. In addition, 24 nurses (48%) experienced pain and discomfort in the oral cavity or teeth during the previous year, and 22 dental nurses (44%) had visited the dentist less than 6 months prior. Moreover, 23 nurses (46%) visited the dentist because of a toothache or other oral problems. The mean oral health behavior score was 4.18 ± 1.17 out of 9, and the mean score of oral and dental problems was 1.06 ± 1.18 out of 10. Regarding behavioral status, 45 dental nurses (90%) demonstrated good behavior, four (8%) exhibited moderate behavior, and one (2%) was categorized as having poor behavior. In terms of reported oral and dental health issues, all 50 nurses (100%) indicated experiencing few problems.

All nurses (100%) reported brushing their teeth. Regarding oral health behavior, 48 nurses (96%) used toothpaste. The frequency distributions of oral and dental health behaviors, as well as problems related to the studied variables, are presented in Tables 1 and 2.

The mean oral health behavior score of single nurses (5.07 ± 1.25) was significantly greater than that of married nurses (3.86 ± 0.97; *p*=0.001). No significant differences were observed in the mean behavior scores based on age, systemic diseases, work experience, workplace, education level, or household income (*p*  > 0.05; Table 1). The frequency distributions of oral and dental health problems are shown in Table 3.

Multivariable regression analysis was used to adjust for potential confounders in oral health behavior and oral problems (Tables 4 and 5).

However, no significant differences were found in the mean scores of oral health problems based on age, systemic diseases, work experience, workplace, family household income, or educational level (*p*-value > 0.05; Table 2).

The results of the Spearman correlation test indicated an inverse relationship between oral health behavior scores and oral health problems among dental nurses (*r* = −0.196); however, this relationship was not statistically significant (*p*-value = 0.172).

## 4. Discussion

### 4.1. Policy Implications

Health policy planners should invest in this number as an icon representing the dental nursing community because the group is capable of contributing to oral and dental health. Improvement in oral health would alleviate the pressure on the healthcare economy to a great extent.

The present survey was conducted to explore the oral health status of female dental nurses who were working in Yazd, Iran. Importantly, all participants were female, reflecting the gender composition of these workplaces. While this homogeneity may restrict broader gender-based analysis, it provided a consistent cohort that reduced gender-based confounding within the scope of this study.

Oral diseases affect an estimated 5.3 million people in all age groups worldwide [15]. With the trend of rising urbanization and a sedentary lifestyle, diseases affecting the oral cavity and teeth will continue to increase. This poses a great burden on public health and community economics [3]. Assessment of oral health status and care is a big challenge in the nursing profession that is usually overlooked [16]. Self-assessed oral health status is based on the general perceptions as described by all individuals concerning their oral health status. Additionally, the scale assessment of global health status can serve as a screening tool for oral health status, complemented by active indicators obtained through oral examination, for monitoring health status in individuals [17]. The index may be helpful in the theoretical understanding of the importance of seeking dental services [18]. It has been assessed in past studies in various communities, including those of hospital nurses, adults, dental nurses, and dental students [1, 18, 19]. The assessment of oral health status within that particular group is important because they have easy access to dental services and relatively high awareness about oral health, imposed by their profession. The index has previously been evaluated among dental students, health workers, the general population, and hospital nurses [1, 18, 19]. In this study, there were no significant differences between oral health behavior and most of the demographic variables, which was consistent with previous studies [18–20]. In this study, the mean score of oral health behaviors was 4.18 out of 9, hence, poor. The finding was similar to An et al.'s [1] study. This suggests that dental hygiene practices may not be as widespread among dental nurses as one might hope. Ninety percent of the responding dental nurses practiced good oral hygiene. This comparison aligns well with the findings from Lawal et al.'s [18] study, and it differs from the result of An et al.'s [1] study at 21.8%. The observed discrepancies in oral health behavior among dental nurses across different populations may be influenced by a variety of contextual factors. Cultural attitudes toward preventive care, oral hygiene procedures, and societal perceptions of dental roles can shape individual behaviors within clinical settings [21, 22]. Systemic differences, such as access to regular education, national public health priorities, and integration of oral health into broader healthcare frameworks, also play a role. These multifaceted influences underscore the need for culturally sensitive interventions and context-specific policy planning when interpreting cross-national findings [1, 19]. Their self-reported score of oral problems was 1 out of 10. This low score may be a factor preventing them from regularly seeking dental services. This finding is consistent with the study conducted by Jahangiry et al. [19], where a majority of adults rated their oral health behavior as good, but only a small percentage reported regular dental visits. Poor oral hygiene may threaten integrity with rising age, generally exacerbating [19]. The average age of the dental nurses in this study was 35.52 years, which was close to Noor et al.'s [17] study and lower than Jahangiry et al.'s [19] study, which was 41.6 years. The oral health behavior score presented no significant difference between the two age groups and supported the findings in some studies. The oral health literacy in some studies increases with increasing age and work experience among nurses [1, 19]. The number of regular dental visits as an index of oral health behavior significantly decreases with increasing age. With the prevalence of oral problems in elderly people, this becomes one of the most important issues to be considered in policy-making [19]. The marital status had a statistically significant difference in oral health behavior. Single dental nurses scored significantly higher in oral health behavior and had fewer oral problems than married dental nurses did. This contrasts with that reported by other previous studies on the topic [1, 20]. Márquez-Arrico et al. [23] claimed that the existence of a relationship between oral health knowledge and educational level in the adult population of Spain was demonstrated.

Mastication difficulty was the main oral health problem in 32% of the participants. The research showed that 44% of the dental nurses had visited a dentist during the last 6 months. In similar studies, this rate was 48.3% in the Chinese nurses and 43.12% in the Iranian adult population, respectively [1, 19]. However, it was lower than that mentioned in the Benzian et al.'s [20] studies.

About 40% of the participants reported good oral health in the current study. In comparison, this was estimated as 50% for the adult community, 50% for dental students, and 20% for Chinese nurses [1, 18, 19]. While some subjects with a very good oral health condition may have sufficient confidence to visit the dentist less frequently than others, there is another contributing factor to this problem [24]. The participants reported that, regarding tooth-cleaning habits, all of them used a toothbrush and 76% of them used dental floss. This may, therefore, suggest that good oral hygiene could account for the low incidence of dental problems. A low score of dental problems, 1 out of 10, could be justified by less frequent dental visits in the participants [25]. In reality, those with good to excellent oral health behavior did not need to regularly visit a dentist. Another study demonstrated that students with more pronounced experience of tooth loss, caries, and supragingival plaque had more negative feelings about their oral health [20]. This problem shows that even though there are dental services, people may have both positive and negative attitudes toward attending a dental visit. These kinds of attitudes are usually not derived from demographic factors [25].

In this study, there was no significant difference in oral health behaviors in terms of family household income and education level.

There are conflicting studies and various studies have reported that sex does not play a part in oral health behavior [1, 19, 20]. A study indicated that women had higher scores for oral hygiene behavior [1]. However, Lawal et al. [18] and Jahangiry et al. [19] did not mention any significant difference between the two genders. In the discrepancy between this study and theirs, it may be stated that people's attitudes toward oral health play a more important role than economic conditions. This result supports the notion that the mere availability of services, even at low costs, is insufficient to establish good oral health habits. Levels of knowledge and attitudes regarding oral health are also key drivers in the development of healthy behaviors. These aspects have to be explored in the future.

### 4.2. Advantages and Limitations of the Study

The study was conducted specifically with female dental nurses. Therefore, any differences related to emotional attitudes between genders were eliminated from the study. On the other hand, no previous study had surveyed informally trained Iranian dental nurses.

There are several drawbacks to the current study. First of all, the current study had a cross-sectional design. Like all other studies with a cross-sectional design, it can only present a snapshot of the problem in a definite period and does not provide information on cause-and-effect relationships. The absence of a control group is a limitation.

The importance of exploring barriers, both structural and behavioral, that may prevent dental nurses from utilizing their access to dental services, despite being embedded in the healthcare ecosystem, is highlighted. Hence, such results need supplementation regarding longitudinal studies and RCTs. There is probably some selection bias. The sampling frame included only two governmental dental clinics in Yazd. Second, while the sample may seem modest, this study was designed to minimize variability and the focused scope contributes to its internal validity.

Third, this study relied on self-reported oral health. This might have resulted in an overestimation or incomplete reflection of actual behaviors, likely due to social considerations.

In light of these considerations, the findings offer value in illuminating oral health patterns within this specific group and can serve as a foundation for larger-scale research.

### 4.3. Suggestion for Future Studies

Incorporating stratified sampling across different clinic types and including male dental professionals in future research would strengthen representativeness and offer more comprehensive insights into occupational oral health disparities.

Qualitative study (consider doing a mixed-methods study): Conducting semistructured interviews with participants to identify psychological, cultural, or logistical barriers that prevent utilization of dental services would be suggested. Future longitudinal or comparative studies that incorporate educated healthcare professionals as controls to assess how educational attainment intersects with oral health behaviors would be proposed.

Given their close interaction with patients, we propose that dental nurses be formally supported through continued professional development programs, including oral health behaviors training modules, to help them serve as credible caregivers in oral health promotion. Future studies involving more diverse samples are encouraged to further validate and expand upon these findings.

## 5. Conclusion

While most participants in our study, a small and homogenous sample of Iranian dental nurses, perceived their oral health status as good, their oral health behavior scores revealed notable shortcomings. These findings underscore the need for targeted educational interventions to address behavioral gaps. Academic courses specifically tailored to dental nurses could serve as an effective tool in promoting evidence-based oral hygiene practices.

## Figures and Tables

**Table 1:** Comparison of the mean oral behavior scores of the dental nurses.

Variables	Number	Mean	SD	*p*-Value	Effect size	95% CI	
						Lower	Upper
Age groups							
≤35	24	4.33	1.16	0.301^a^	0.283^c^	−0.374	0.963
>35	26	4.03	1.18				
Marital status							
Single	13	5.07	1.25	0.0001^a^	0.953^c^	0.529	1.894
Married	37	3.86	0.97			0.404	2.019
Work experience							
<12 years	26	4.34	1.26	0.321^a^	0.272^c^	−0.320	1.012
≥12	24	4	1.06			−0.316	1.008
Systemic disease							
Yes	2	4	1.41	0.877^a^	0.042^c^	−1.905	1.530
No	48	4.18	1.17			−11.503	11.128
Workplace							
Dental school	35	4.28	1.27	0.514^a^	0.177^c^	−0.375	1.080
Khatamolanbia Clinic	15	3.93	0.88			−0.282	0.987
Family household income							
<10 million tomans	19	1.21	1.51	0.629^b^	0.023^d^	0.481	1.939
10–15	19	0.94	0.91			0.508	1.386
>15	12	1	1.04			0.336	1.663
Educational level							
Post-diploma and lower diploma	20	4.05	1.00	0.134^b^	0.043^d^	0.707	2.459
Bachelor's degree	24	4.04	1.16			0.154	2.131
Master's degree and higher	6	5.16	1.47			50−0.261	1.498 0.595

^a^Mann‒Whitney *U* test.

^b^Kruskal‒Wallis test.

^c^Cohen's *d* effect size for Mann–Whitney *U* test.

^d^Eta-squared effect size for Kruskal–Wallis *H* test.

**Table 2:** Comparison of the mean scores of oral health problems among dental nurses.

Variables	Number	Mean	SD	*p*-Value	Effect size	95% CI	
						Lower	Upper
Age groups							
≤35 years	24	0.79	0.93	0.168^a^	0.374^c^	−1.180	0.330
>35	26	1.30	1.34			−1.172	0.325
Marital status							
Single	13	0.46	0.51	0.04^a^	0.569^c^	−1.548	−0.068
Married	37	1.27	1.28			−1.322	−0.294
Work experience							
<12 years	26	0.96	1.24	0.343^a^	0.255^c^	−0.884	0.473
≥12	24	1.16	1.12			−0.881	0.471
Systemic disease							
Yes	2	0.50	0.70	0.529^a^	0.169^c^	−2.312	1.145
No	48	1.080	1.19			−4.807	3.640
Workplace							
Dental school	35	1.05	1.23	0.849^a^	0.051^c^	−0.752	0.733
Khatamolanbia Clinic	15	1.06	1.09			−0.729	0.710
Family household income							
<10 million tomans	19	1.21	1.51	0.929^b^	0.043^d^	0.481	1.939
10–15	19	0.94	0.91			0.508	1.386
>15	12	1	1.04			0.336	1.663
Educational level							
Post-diploma and lower	20	1.40	1.23	0.032^b^	0.104^d^	0.707	2.459
Bachelor's degree	24	1	1.17			0.154	2.131
Master's degree and higher	6	0.16	0.40			0.501 −0.261	1.498 0.595

^a^Mann‒Whitney *U* test.

^b^Kruskal‒Wallis test.

^c^Cohen's *d* effect size for Mann–Whitney *U* test.

^d^Eta-squared effect size for Kruskal–Wallis *H* test.

**Table 3:** Frequency and percentage of oral health problems experienced during the previous 12 months.

Problem type	Frequency	
	Yes *N* (%)	No *N* (%)
Mastication difficulty	16 (32)	34 (68)
Speech/word pronouncing difficulties	1 (2)	49 (98)
Felt tense due to teeth or mouth	11 (22)	39 (78)
Felt embarrassed due to the appearance of my teeth	14 (28)	36 (72)
Smiling avoidance because of concerns about teeth or mouth	1 (2)	49 (98)
Sleep interruption	2 (4)	48 (96)
Days off work	0 (0)	50 (100)
Difficulty doing usual activities	0 (0)	50 (100)
Felt less tolerant of a spouse or close people	3 (6)	47 (94)
Reduced tolerance of spouse or close friends	5 (10)	45 (90)

**Table 4:** Multiple regression analysis for explanatory variables in oral health behavior.

Explanatory variables	Coefficient	Std. error	95% confidence interval		*p*-Value
			Lower	Upper	
Age groups					
≤35 years	Reference				
>35	0.305	0.4246	−0.528	1.137	0.473
Marital status					
Single	Reference				
Married	−1.384	0.4550	−2.275	−0.492	0.002
Work experience					
<12 years	Reference				
≥12	0.274	0.4543	−0.617	1.164	0.547
Family household income					
<10 million tomans	Reference				
10–15	0.232	0.3567	−0.467	0.931	0.515
>15	0.072	0.4020	−0.716	0.860	0.857
Educational level					
Post-diploma and lower	Reference				
Bachelor's degree	0.097	0.3540	−0.597	0.790	0.097
Master's degree and higher	0.606	0.5524	−0.476	1.689	0.272

**Table 5:** Multiple regression for explanatory variables in oral problems.

Explanatory variables	Coefficient	Std. error	95% confidence interval		*p*-Value
			Lower	Upper	
Age groups					
≤35 years	Reference				
>35	0.343	0.4479	−0.535	1.221	0.444
Marital status					
Single	Reference				
Married	0774	0.4799	−0.166	1.715	0.107
Work experience					
<12 years	Reference				
≥12	−0.739	0.4791	−1.678	0.201	0.123
Family household income					
<10 million tomans	Reference				
10–15	−0.177	0.3762	−0.915	0.560	0.637
>15	0.023	0.4240	−0.854	0.808	0.957
Educational level					
Post-diploma and lower	Reference				
Bachelor's degree	−0.450	0.3734	−1.182	0.281	0.227
Master's degree and higher	−1.066	0.5826	−2.208	0.075	0.067

*Note:* In terms of oral health problems, there was a significant difference in the mean behavior score between single dental nurses (0.46 ± 0.51) and married nurses (1.27 ± 1.28; *p*-value = 0.001).

## Data Availability

All data analyzed during this study are included in this published article. Additional data/files may be obtained from the corresponding author upon reasonable request.

## Nomenclature

OHQ: Oral health self-assessment questionnaire
WHO: World Health Organization.
